# Antiplasmodial activity of *Indigofera spicata* root extract against *Plasmodium berghei* infection in mice

**DOI:** 10.1186/s12936-017-1853-5

**Published:** 2017-05-16

**Authors:** Eshetie Melese Birru, Mestayet Geta, Abyot Endale Gurmu

**Affiliations:** 1 Department of Pharmacology, School of Pharmacy, College of Medicine and Health Sciences, University of Gondar, Chechela Street, Lideta Subcity kebele 16, P.O.box 196, Gondar, Ethiopia; 2 Department of Pharmacognosy, School of Pharmacy, College of Medicine and Health Sciences, University of Gondar, Chechela Street, Lideta Subcity kebele 16, P.O.box 196, Gondar, Ethiopia

**Keywords:** Malaria, Herbal medicine, *Indigofera spicata*, *Plasmodium berghei*, In vivo

## Abstract

**Background:**

In addition to pharmacovigilance and pharmaco-economic concerns, resistance to anti-malarial medicines has been documented in all classes of anti-malarials and this is further worsened by resistance to common insecticides by malaria vector, which is a major threat to malaria control. As a means of facing the challenges of searching for new anti-malarial agents, the current study focused on evaluation of anti-malarial activity of root extract of *Indigofera spicata*.

**Methods:**

Chloroquine-sensitive rodent malaria parasite, *Plasmodium berghei* (ANKA strain) was used to infect the Swiss Albino mice in 4-day suppressive and curative models. The crude hydromethanolic root extract of *I. spicata* at 200, 400 and 600 mg/kg doses was administered to a group of five mice. Important parameters, such as level of parasitaemia, packed cell volume (PCV), survival time, and body weight were determined and the significance of the differences between mean values of the five groups was analysed by one-way ANOVA followed by post hoc Tukey’s Multiple Comparison test.

**Results:**

In both the suppressive and curative models, 400 and 600 mg/kg doses of the extract suppressed the level of parasitaemia significantly (p < 0.001) compared to the vehicle-treated groups, 34.93 and 53.42%, respectively. However, only the mice which were treated with the 600 mg/kg dose of the extract had significant difference in their mean survival time. In other parameters, namely PCV and mean body weight, there was no statistically significant difference between the extract-treated groups when compared to the negative control.

**Conclusions:**

This study revealed that the root extract of *I. spicata* possesses anti-malarial activity and necessitates further scientific validation.

## Background

According to the World Health Organization (WHO), in 2012 there was an estimated 207 million cases of malaria and 627,000 malaria deaths worldwide. The majority of the estimated cases (80%) and deaths (90%) occur in sub-Saharan Africa. Furthermore, most (77%) of the deaths occur in children under 5 years of age, and mainly the deaths were due to *Plasmodium falciparum*. However, *Plasmodium vivax* is increasingly recognized as a cause of severe malaria and death. About 9% of estimated cases globally are due to *P. vivax*, although the proportion outside the African continent is 50% [1, 2]. In the eastern and southern African regions, an estimated 30% of all recorded deaths during pregnancy are attributed to malaria infection [3].

Malaria is number one health problem in Ethiopia and it is the main cause of morbidity and mortality. According to the 2016 WHO Report, there were over 1.8 million confirmed malaria cases in Ethiopia in 2015 [4]. The economic impact of malaria is very significant, as the country’s economy is based on agriculture and peak malaria transmission coincides with the planting and harvesting season. Historically, malaria has forced people to inhabit the less agriculturally productive highlands. About 75% of the country is malarious, with about 68% of the country’s total population living in areas at risk of malaria [5, 6].

The malaria vector, the female *Anopheles mosquito*, which has developed resistance against insecticides, such as dichlorodiphenyltrichloro-ethane (DDT), and chemoprophylaxis has not often yielded the expected results. Additionally, the disease-causing protozoans have developed resistance against most of the drugs currently used to treat malaria. New, highly effective anti-malarial drug candidates, based on new mechanisms of action or with new structures, are urgently needed to overcome the problem of rapid emergence of drug resistance and achieve long-term clinical efficacy [7, 8].

Traditional preparations have been the main source of treatment of malaria in Africa and other continents where the disease is endemic and resources are limited [9]. They have, in the past, been the source of some of the most successful anti-malarial agents, such as the quinolines and the endoperoxide artemisinin. One of the areas to search for new anti-malarials is the traditionally claimed anti-malarial plants from the African flora. It has been claimed that about 80% of the Ethiopian population rely on medicinal plants for treating various illnesses, including malaria [10]. However, traditional medicine is not without its limitations, such as lack of science-based efficacy and safety evaluations [11]. Although several plants that have potential anti-malarial properties have been studied, there still exists innumerable potentially useful medicinal plants awaiting evaluation and exploitation for therapeutic applications against various groups of pathogens [7, 8]. Furthermore, the possibility that some chemicals could serve as a prodrug and immunomodulatory agents in managing infectious disease will make this study justifiable when conducted in vivo anti-malarial models [12, 13].

The root of *Indigofera spicata* have been used orally in the treatment of malaria [14, 15] and traditional healers use the plant material locally. Yet systemic pharmacological studies have not been reported to support its claim. This study was designed to evaluate the in vivo anti-malarial effect of root extract of *I. spicata.*

## Methods

### Collection and preparation of plant materials

Fresh roots of *I. spicata* were collected from the surrounding area of Shawra town, South West Gondar Province, North West Ethiopia in December, 2014. The plant was botanically identified and a voucher specimen is already deposited in the Department of Biology, National Herbarium, Addis Ababa University.

### Animals used

Male Swiss albino mice were used, 22–30 g weight and 6–8 weeks old, from the Department of Pharmacology, College of Medicine and Health Sciences, University of Gondar animal house. The housing and feeding conditions were maintained as per recommended standards. All mice were acclimatized to the working environment 1 week before the beginning of the experiment. They were also handled according to the international animal care and welfare guidelines [16, 17].

### Parasites inoculation

Chloroquine-sensitive *Plasmodium berghei* (ANKA strain) was used for induction of malaria in experimental mice. Mice previously infected with *P. berghei* were used as donor. The donor *P. berghei*-infected mice were obtained from Aklilu Lemma Institute of Pathobiology, Addis Ababa University, Ethiopia. The parasites were subsequently kept alive by continuous serial intraperitoneal (IP) passage of blood from donor mouse to uninfected mouse on a weekly basis.

### Drugs and reagents

Methanol (Avishkar Lab Tech Chemicals, India), Giemsa (ScienceLab, USA), chloroquine (Addis Pharmaceuticals Factory, Ethiopia), trisodium citrate (Deluxe Scientific Surgico, India), Tween-80 (Avishkar Lab Tech Chemicals, India), ketamine (Rotexmedica, Germany), normal saline (Epharm, Ethiopia), hydrochloric acid (Supertek Chemicals, India), chloroform (Avishkar Lab Techchemicals, India), sulfuric acid (Supertek, India), nitric acid (Supertek, India), acetic anhydride (Central Drug House, India), ferric sulfate (BDH Ltd, UK), ferric chloride (Fisher Scientific Co, USA), lead acetate (BDH Ltd, UK), benzene (Nice laboratory reagent, India), Mayer’s reagent (Avishkar Lab Tech Chemicals, India), Wagner’s Reagent (BDH Ltd, UK) were used. All reagents were analytically graded and procured from certified local and international suppliers.

### Extraction of plant material

The fresh roots of *I. spicata* were thoroughly washed with distilled water to remove dirt and soil, and dried under shade and optimal ventilation for 2 weeks. The dried roots were further chopped into small pieces and reduced to powder using an electronic miller. The powdered roots of the plant were weighed before maceration with 80% methanol. The coarsely powdered roots were subjected to maceration extraction procedure using 80% methanol (for 72 h at room temperature) as a menstruum and this was done three times. The respective extract was filtered using Whatman No-1 filter paper and the solvent was evaporated in an oven under reduced pressure at 40 °C. Finally the dried extract was stored at 4 °C in refrigerator until used.

### Infecting, grouping and dosing of animals

Blood was taken from a donor mouse with approximately 30% parasitaemia and diluted in physiological saline to 5 × 10^7^ parasitized erythrocytes per ml. Swiss albino mice (male) weighing 24–29 g were infected with 0.2 ml blood (1 × 10^7^ *P. berghei* parasitized erythrocytes*)* intraperitoneally and randomly divided into three test groups and two control groups (each for 25 mg/kg chloroquine [18] as a standard drug and normal saline as a negative control). The extract doses 200, 400, 600 mg/kg were determined based on the previous acute toxicity evaluation reports [19] and preliminary anti-malarial activity evaluation done on this extract. For all animals, volume of administration was 1 ml/100 g [16] and as the plant material is traditionally used [14] to treat malaria, the oral route of administration was used in all cases [20, 21].

### 4-day suppressive test

It is the most widely used preliminary test, in which the efficacy of a compound is assessed by comparison of blood parasitemia and mouse survival time in treated and untreated mice [22]. Treatment of infected mice was started after 3 h of infection on day 0 and continued daily for 4 days (i.e., from days 0–3). On the fifth day (day 4) blood samples were collected from tail snip of each mouse. Thin smears were prepared and stained with 10% Giemsa solution. Then, each stained slide was examined under the microscope with an oil immersion objective of 100× magnification power to evaluate the percent suppression of each extract with respect to the control groups. Average percent parasitaemia and suppression was calculated by using the following formula [19, 23].

$$\% {\text{ Parasitaemia }} = \frac{{{\text{Number of parasitized red blood cells }}\left( {\text{RBC}} \right)}}{\text{Total number of RBC count}} \times { 1}00$$

$${\text{\% Suppression}} = \frac{{\left( {\% {\text{ Parasitemia of negative control}} - \% {\text{ Parasitaemia of treated group}}} \right)}}{{\% {\text{ Parasitaemia of negative control}}}} \times 100$$

### Curative test

Curative test is the evaluation of the efficacy of a chemical substance against an established malaria infection by using level of parasitaemia suppression and survival time as important parameters. Thus, evaluation of curative anti-malarial potential of the extract was done by using a method described by Ryley and Peters [21]. On the first day, standard inoculum of 1 × 10^7^ *P. berghei*-infected RBCs were injected intraperitoneally into 25 Swiss albino mice. Seventy-two hours later the mice were randomly divided into five groups of five mice each. Subsequently different doses of the extract (200, 400 and 600 mg/kg/day), chloroquine phosphate (25 mg/kg/day) and the vehicle (10 ml/kg/day) were given orally to the respective groups for 5 consecutive days.

For each mouse, thin blood films stained with 10% Giemsa was prepared from tail blood of each mouse daily for 5 consecutive days to monitor the levels of parasitaemia. Starting from the date infection (day 0) the mice were observed for 15 days and any death that occurred during this period was recorded and used to determine the mean survival time. Average percentage parasitaemia suppression will be calculated:

$${\text{A }} = \left( {{\text{D}} - {\text{E}}} \right) \times { 1}00\,{\text{D}}$$

where, A = average percentage suppression of parasitaemia, D = average percentage parasitaemia before treatment and E = average percentage parasitaemia after treatment.

### Determination of packed cell volume

Determination of packed cell volume (PCV) was measured to predict the effectiveness of the test extracts in preventing haemolysis resulting from increasing parasitaemia associated with malaria. Blood was collected from the tail of each mouse in heparinized microhematocrit capillary tubes by filling three-quarters of its volume. The tubes were sealed by sealant and placed in a microhematocrit centrifuge with the sealed ends outwards. The blood was centrifuged at 12,000 rpm for 15 min. The PCV of each mouse was then measured before infection and on day 4 after infection using the formula:

$${\text{PCV }} = {\text{ Volume of erythrocytes in a given volume of blood}}/{\text{total blood volume }} \times { 1}00$$

### Determination of level of Parasitaemia

Tail blood smearing of each mouse was made for both the suppressive and curative tests. After fixation with absolute alcohol and staining with 10% Giemsa at pH 7.2 for 15 min, the slides were washed gently with distilled water followed by air drying at room temperature. By using Olympus microscope oil immersion objective of 100× magnification power, the number of parasitized erythrocytes in random fields of the microscope was counted. Three separate fields on each of the slides were used to calculate percent parasitaemia and percent suppression using the following formula, respectively.

$$\% {\text{ Parasitaemia }} = {\text{ Number of parasitized RBC}}/{\text{Total number of RBC }} \times { 1}00$$

### Determination of mean survival time

From the time of infection until death, mortality of each mouse was monitored and recorded regardless of the group in which the mouse was allocated throughout the follow-up period (15 days). Mean survival time of mice of each group was determined using the following formula:

$${\text{MST}} = \frac{\text{Sum of survival time of all mice in a group (days)}}{\text{Total number of mice in that group}}$$

### Determination of body weight

For Peter’s test, body weight of each mouse was measured before infection (day 0) and on day 4 using sensitive electronic balance.

### Data quality control

Each of the microscopic slides was coded and the measurements were determined blindly by a laboratory technician. In addition, for greater reliability each measurement was done in triplicate.

## Ethical clearance

The study protocol was ethically reviewed by the Institutional Review Board of the University of Gondar. All protocols were performed in accordance with the international animal care and welfare guidelines [16].

### Statistical analysis

All data values are expressed as mean ± SEM (standard error of means) for five mice per group. Statistical analyses were carried out by using SPSS Statistical software version-21. Statistical significance of mean of parasitaemia suppression, weight, PCV, and survival time differences between groups was computed by one-way ANOVA, followed by post hoc Tukey’s Multiple Comparison Test. *p* value of less than 0.05 was considered statistically significant.

## Results

### 4-day suppressive test

As it is shown in Table 1, compared to the vehicle treated group, mice treated with 200, 400 and 600 mg/kg doses of the plant extract had 17.12, 34.93 and 53.42% suppression of parasite level, respectively. Comparison between the extract-treated groups revealed that mice treated with 600 mg/kg dose had statistically significant (p < 0.001) difference compared to those treated with 200 mg/kg dose. However, level of parasitaemia suppression due to chloroquine was found significantly (p < 0.001) higher than all the extract dose treated groups. Considering mean survival time, among extract-dose treated groups, only those treated with 600 mg/kg had statistically significant longer period of survival compared to the vehicle-treated group. Comparison among the extract-treated groups demonstrated that only those treated with 600 mg/kg had statistically significant mean survival time in reference to 200 mg/kg treated groups (p < 0.001).

**Table 1:** Four-day suppressive test: activity of hydroalcoholic crude root extract of *Indigofera spicata*on level of parasitemia and survival time of mice infected with *Plasmodium berghei*

Group	Level of parasitemia	Percent suppression	Survival date
NS	29.2 ± 1.85	–	6.6 ± 0.89
200 mg/kg IS	24.2 ± 1.24	17.12	7.2 ± 0.84
400 mg/kg IS	19.0 ± 1.22^a,3^	34.93	8.0 ± 0.71
600 mg/kg IS	13.6 ± 1.29^a,3,b,3^	53.42	8.8 ± 0.84^a,2,b,1^
25 mg/kg CQ	0.6 ± 0.4^a,3,b,3,c,3,d,3^	89.04	>14.6 ± 0.89^a,3,b,3,c,3,d,3^

Data are expressed as mean ± SEM; n = 5

IS, *Indigofera spictata*; CQ, chloroquine

^a^Compared to control

^b^To 200 mg/kg

^c^To 400 mg/kg

^d^To 600 mg/kg

^1^p < 0.05

^2^p < 0.01

^3^p < 0.001

In the 4-day suppressive test, none of the extract dose-treated groups was significantly protected from PCV reduction. However, the standard drug induced significant protection against PCV reduction compared to both the normal saline and the 200 mg/kg extract dose-treated groups (p < 0.01). In addition, neither the extract doses nor the standard drug-treated group demonstrated statistically significant difference in body weight compared to the control group (Table 2).

**Table 2:** Effect of hydroalcoholic crude root extract of *Indigofera spicata* on packed cell volume and body weight of infected mice in the 4 day suppressive test

Group	PCV			MBW		
	D0	D4	% change	D0	D4	% change
NS	53.56 ± 0.81	49.92 ± 1.34	−6.80	26.28 ± 0.51	24.36 ± 0.64	−7.31
200 mg/kg IS	53.08 ± 0.77	50.54 ± 1.91	−4.79	26.48 ± 1.2	25.42 ± 1.01	−4.00
400 mg/kg IS	53.56 ± 0.79	52.32 ± 1.25	−2.32	26.26 ± 0.77	25.68 ± 0.6	−2.21
600 mg/kg IS	53.0 ± 0.55	52.28 ± 0.76	−1.36	25.96 ± 1.13	25.42 ± 0.91	−2.08
25 mg/kg CQ	53.68 ± 0.72	54.22 ± 1.22^a,2,b,2^	1.01	26.84 ± 0.97	27.32 ± 1.18	1.79

Data are expressed as mean ± SEM; n = 5

IS, *Indigofera spictata*; CQ, chloroquine; MBW, mean body weight

^a^Compared to control

^b^To 200 mg/kg

^c^To 400 mg/kg

^d^To 600 mg/kg

^1^p < 0.05

^2^p < 0.01

^3^p < 0.001

### Curative activity evaluation

The plant material suppressed the parasite level of mice by 58.18, 78.04 and 85.88% at day 7 compared to the normal saline-treated group, while the suppression induced by the standard drug was 100%. In parallel, compared to the negative control at day 7, those treated with the extract (all doses) and chloroquine had significantly (p < 0.001) reduced level of parasitaemia. Furthermore, like the positive control on day 7, both 400 and 600 mg/kg extract dose-treated groups had significantly (p < 0.001) controlled level of parasitaemia compared to the 200 mg/kg extract dose-treated groups. Except the 600 mg/kg dose, none of the extract doses suppressed the parasite level significantly compared to the normal saline at day 4. In the next days, all the extract doses reduced the level of parasitaemia considerably to various extent compared to the control group.

In mean survival time, only the 400 mg/kg (p < 0.05) and 600 mg/kg (p < 0.01) extract doses substantially prolonged survival time compared to normal saline. However, the mean survival time of mice treated with chloroquine was significantly (p < 0.001) higher than not only the negative controls but also the extract dose-treated groups (Table 3).

**Table 3:** Effect of crude hydromethanolic root extract of *Indigofera spicata* on level of parasitemia and survival time of mice infected with *P. berghei* in curative test

Group	Level of parasitemia						Survival date
	D3	D4	D5	D6	D7	% change	
NS	18.4 ± 1.78	24.8 ± 1.8	30.4 ± 0.68	40.75 ± 1.65	51 ± 2.00	–	6.2 ± 0.37
200 mg/kg IS	17.6 ± 1.72	22 ± 1.67	22.6 ± 1.89^a,2^	20.6 ± 1.5^a,3^	21.7 ± 1.76^a,3^	58.18	6.8 ± 0.84
400 mg/kg IS	16.2 ± 1.66	18.8.4 ± 1.16	16.2 ± 0.74^a,3,b,1^	15.8 ± 1.36^a,3,b,2^	13.3 ± 1.59^a,3,b,3^	74.12	7.8 ± 0.37^a,1^
600 mg/kg IS	18.8 ± 1.16	18.2 ± 1.56^a,1^	15.4 ± 2.09^a,3,b,2^	10.4 ± 1.21^a,3,b,3^	10.2 ± 0.58^a,3,b,3^	80.00	8.2 ± 0.84^a,2^
25 mg/kg CQ	16.6 ± 1.96	11.4 ± 1.21^a,3,b,2,c,1,d,1^	4 ± 0.45^a,3,b,3,c,3,d,3^	0.4 ± 0.25^a,3,b,3,c,3,d,3^	0.00^a,3,b,3,c,3,d,3^	100	>15^a,3,b,3,c,3,d,3^

Data are expressed as mean ± SEM; n = 5

IS, *Indigofera spictata*; CQ, chloroquine

^a^Compared to control

^b^To 200 mg/kg

^c^To 400 mg/kg

^d^To 600 mg/kg

^1^p < 0.05

^2^p < 0.01

^3^p < 0.001

## Discussion

In addition to the claimed traditional use, an advantage of the mouse model is that it may detect compounds requiring bioactivation and/or which have immunomodulatory activity [21]. More often, in the early in vivo anti-malarial activity evaluation, use of *P. berghei* as an infecting parasite is recommended. Similarly, the 4-day suppressive model and the Raye’s test are also widely acceptable in order to evaluate the anti-malarial potential of natural or synthetic products on early infection and late infections, respectively [19, 21].

Anti-malarial test materials with more than 30% suppressive effect on the level of parasitaemia [7, 24] or that can prolong the survival date of treated mice [25] compared to the control group are often considered effective in standard screening tests, which is in agreement with this study finding. As is depicted in Table 1, in the 4-day suppressive test the 400 and 600 mg/kg plant extract doses had significant anti-malarial activity (p < 0.05) in a dose dependent manner which was further demonstrated by the mean survival time of the mice. Similarly, many previously reported plant materials have shown dose-dependent anti-malarial effect [26]. In the 4-day suppressive model, none of the extract dose-treated groups of mice illustrated significant change in level of PCV compared to the negative control and that is why this evaluation parameter was not considered in the curative test of the plant material. Lack of significant PCV protection from the extract doses might be due to the presence of saponins in the crude extract, which are known to cause haemolysis by increasing the permeability of the plasma membrane [27, 28]. Active anti-malarial agents have been expected to protect weight loss associated with malaria infection but in this study the protective effect of the extract doses on loss of body weight was not found to be significant. The discrepancy might be explained by the imbalance of the protective effect of the extract and the cumulative pathophysiologic changes associated with the infection. 

In the curative test, the significant (p < 0.001) suppression of level of parasitaemia across all doses was also revealed in a dose-dependent manner. Accordingly, this is confirmation of the plant material having effective antiplasmodial activity in the late stages of the infection. Hopefully, a plant with such anti-malarial activity at crude extract level may serve as a promising lead compound for further optimization and development of new therapeutic agents.

Complementing the demonstrated parasitaemia suppression of the plant material, this study also revealed that animals that were treated with the extract live longer than animals treated with the vehicle. Even if it was not statistically significant, there was similar reflection in terms of PCV and body weight parameters. This might be explained by the presence of various secondary plant metabolites in the crude extract that may have a complex biochemical effect and lack of very strong anti-malarial activity that could ameliorate all pathologic changes. Furthermore, this might be associated with the impurity of the extract and if the active principle(s) were isolated and/or optimized the activity of the plant material might statistically sound in all evaluation parameters.

Substances with antioxidant activity have known antimicrobial activity, i.e., associated with the elevated level of oxidative stress in a person who is being infected. As it was confirmed in previous reports [29], this plant has antioxidant activity which might support its antidiabetic, anticancer, antidiarrhoeal activities [19, 30] and the current anti-malarial activity of the extract. Furthermore, the antiplasmodial activity of the plant material is supposed to be due to the presence of secondary plant metabolites, such as alkaloids, flavonoids and tannins. Those metabolites may work independently or synergistically [31, 32].

In summary, according to the anti-malarial activity classification of Deharo et al. [33], the methanolic crude extract of the root of *I. spicata* showed moderate anti-malarial activity in both the 4-day suppressive and curative models.

## Conclusions

The finding of this study supports the claimed traditional use of *I. spicata* in treating malaria. From this, there may be a possibility of developing new anti-malarial lead compound with rigorous molecular mechanism and characterization studies.

## Abbreviations

IRBC: infected red blood cells
NS: normal saline
PCV: packed cell volume
WHO: World Health Organization
